# Aucubin prevents steroid‐induced osteoblast apoptosis by enhancing autophagy via AMPK activation

**DOI:** 10.1111/jcmm.16954

**Published:** 2021-10-06

**Authors:** Chen Yue, Hongting Jin, Xue Zhang, Wuyin Li, Deli Wang, Peijian Tong, Youwen Liu, Zhen Tan

**Affiliations:** ^1^ Department of Orthopedic Luoyang Orthopedic Hospital of Henan Province Luoyang China; ^2^ Institute of Orthopaedics and Traumatology The First Affiliated Hospital of Zhejiang Chinese Medical University Hangzhou China; ^3^ Department of Bone and Joint Surgery Peking University Shenzhen Hospital Shenzhen China

**Keywords:** apoptosis, aucubin, autophagy, osteoblast, SONFH, steroid

## Abstract

Steroid‐induced osteoblast apoptosis is a crucial pathological process in steroid‐induced osteonecrosis of the femoral head (SONFH). Autophagy can resist apoptosis and AMPK plays an important role in autophagy regulation. Aucubin from the small tree Eucommia ulmoides Oliv., which has a long history of use in orthopaedics and traumatology in Asian medicine, can promote bone formation, but whether it can slow or prevent steroid‐osteoblast apoptosis is unclear. Therefore, we investigated the pathogenesis of SONFH and how the osteoblast responds to aucubin under the dexamethasone stimulation. In human femoral head osteonecrosis specimens, we found that the autophage and apoptosis level were increased, and the AMPK signalling was crucial to autophagy. We observed that aucubin could prevent dexamethasone‐induced apoptosis in osteoblasts by enhancing the level of autophagy. Further, we confirmed that the regulatory effect of aucubin on autophagy and apoptosis was achieved by activating AMPK signalling. We have demonstrated a mechanism of disease progression and shown that aucubin could enhance autophagy through AMPK signalling to prevent osteoblast apoptosis. These findings provide a basis for the further investigation of the potential therapeutic role of aucubin in the SONFH.

## 1 INTRODUCTION

As a result of their powerful anti‐inflammatory and immunosuppressive ability, steroids such as dexamethasone and prednisone are commonly used to treat systemic lupus erythematosus, nephrotic syndrome and other autoimmune disease.^1^, ^2^ However, excessive steroids usage is a common cause of osteonecrosis of the femoral head.^3^ The pathological mechanism of steroid‐induced osteonecrosis of the femoral head (SONFH) is very complex, and it has been confirmed that steroids target a variety of cells including mesenchymal stem cell, osteoblast, osteoclast, osteocyte and endothelial cell.^4^ Osteoblast is the main functional cell of bone formation, which directly affects the balance of bone metabolism. Excessive steroids can induce osteoblast apoptosis, impairing bone tissue repair, and this decreased ability to repair bone may eventually progress to SONFH.^5^, ^6^ Recent evidence indicates that autophagy is involved in the regulation of steroid‐induced osteoblast apoptosis, which has opened a new avenue of SONFH therapy.^7^, ^8^

Autophagy is a complex biological process that involves degrading dysfunctional cellular components and clearing damaged proteins through lysosomal pathways, providing nutrient recycling and generating energy; it is an important process in cell growth, function and homeostasis maintenance.^9^, ^10^ Research over the past 10 years has led to the idea that autophagy can help maintain cell homeostasis and normal function in the presence of external insults.^11^, ^12^, ^13^, ^14^

Autophagy is complex and involves many signalling pathways. AMP‐activated protein kinase (AMPK) is a serine/threonine protein kinase, considered the master sensor of cell energy and metabolism. It is sensitive to a broad spectrum of stressors, especially those that alter cellular energy status.^15^, ^16^ AMPK has critical roles in regulating cell growth and survival, and AMPK activation has recently been shown to provoke cytoprotective autophagy to recycle cellular components.^17^, ^18^, ^19^

With the successful application of the traditional Chinese medicine artemisinin to treat malaria,^20^ researchers are increasingly focusing on understanding and harnessing the active ingredients in natural herbs.^21^, ^22^ Known as a useful herb for strengthening bone, *Eucommia ulmoides* Oliv. has been widely used to treat many kinds of orthopaedic diseases in Asian countries for thousands of years.^23^, ^24^ The iridoid glycoside aucubin (C_15_H_22_O_9_), the principle bioactive component of *Eucommia ulmoides* Oliv., has been reported to exert several pharmacological effects against inflammation, oxidation and bone loss.^23^, ^24^, ^25^ In fact, aucubin has recently been shown to prevent apoptosis by regulating the level of autophagy in several disease models.^26^, ^27^ However, little is known about whether and how aucubin might influence steroid‐induced autophagy and apoptosis in osteoblast.

Therefore, in this study, we wished to clarify the role of AMPK signalling in disease progression, and to determine whether aucubin could prevent steroid‐induced osteoblast apoptosis by modulating autophagy via AMPK activation. We first confirmed that the levels of *p*‐AMPK, autophagy and apoptosis are elevated in human SONFH specimens, suggesting that AMPK signalling participates in autophagy and apoptosis in the process of SONFH. We therefore considered AMPK as a potential molecular target and found that aucubin can increase AMPK activation to promote autophagy and prevent steroid‐induced osteoblast apoptosis. Our data suggest that aucubin may be a therapeutic medicine in the prevention and treatment of SONFH.

## 2 MATERIALS AND METHODS

### 2.1 Human SONFH specimens and histological evaluation

All participants were recruited between April 2020 and July 2020 from our hospital and provided informed consent according to institutional guidelines. Human femoral head tissue was harvested from patients with SONFH (*n* = 5) and control patients with hip osteoarthritis undergoing total hip arthroplasty (*n* = 5). When obtaining the specimens during operation, approximately 15% of the anterior and posterior of the femoral head in the coronal position was removed to facilitate the penetration of formalin. The use of these specimens for research purposes was approved by the institutional review board of Luoyang Orthopedic Hospital of Henan Province.

Femoral head samples were fixed in 10% neutral formalin for 4 weeks. After fixation, samples were decalcified in 0.5 M EDTA for six months. The bone section preparation was performed as previously described.^28^ The sections were stained with hematoxylin and eosin (H&E) to evaluate the necrosis of bone in the femoral head. Photomicrographs from each section were obtained utilizing a digital camera (Olympus). The immunofluorescence staining was incubated with antibodies against osterix (1:50 dilution, Abcam ab209484, USA), cleaved caspase‐3 (1:50 dilution, CST #9664, USA), Beclin‐1 (1:50 dilution, Abcam ab210498, USA), *p*‐AMPK (1:50 dilution, CST #2535, USA) and LC3B (1:50 dilution, CST #83506, USA).

### 2.2 Pre‐osteoblast MC3T3‐E1 cells

Murine pre‐osteoblast MC3T3‐E1 cells were a gift from Sichuan University's School of Basic and Forensic Medicine. They were cultured in Dulbecco's Modified Eagle Medium (DMEM, Gibco #11054‐020, USA) supplemented with 10% foetal bovine serum (FBS, Hyclone #SH30071.03, USA), 100 U/ml penicillin‐100 μg/ml streptomycin solution (Hyclone #C838W20, USA) and 1 mM glutamine (Sigma‐Aldrich #G8540, USA). Cultures were maintained in a humidity incubator (Thermo Fisher Scientific #3110, USA) with 5% CO_2_ at 37°C. Culture medium was changed every 2–3 days.

After the cells were grown to 80% confluence, they were treated with dexamethasone (1 µM), aucubin (Macklin Biochemical Technology Co. Ltd A890229), autophagy inhibitor 3‐MA (Selleck #S2767, USA) or short hairpin RNA against AMPK (shAMPK, GenePharma #D02001, China) treatments in various combinations for subsequent experiments.

### 2.3 Cell viability assay

The Cell Counting Kit‐8 (Beyotime C0038) was used to test the cell viability according to the manufacturer's protocol. Briefly, the cells were seeded on 96‐well plates at 1 × 10^4^ cells/well overnight. After 48 h of intervention, the CCK‐8 solution was added to each well in a 1:10 dilution in the culture medium. After incubation for 4 h at 37°C and 5% CO_2_ in a humidified atmosphere, absorbance was measured at 460 nm by a microplate reader (Molecular Devices).

### 2.4 AMPK Knock‐down lentiviral transduction

Lentiviruses encoding human AMPK short hairpin RNA (LV‐shAMPK) or control shRNA (LV ‐ shcontrol) were purchased from GenePharma (GenePharma #D02001, China). The lentivirus expressed green fluorescent protein (GFP) and blasticidin S deaminase. Thus, 10 μg/ml of blasticidin S (Yeasen #60218ES10, China) was used to select HEK293 for expressing LV‐shAMPK, and then the supplement containing LV‐shAMPK particles was incubated with 70%–80% confluent of MC3T3‐E1 cells in DMEM supplemented with heat‐inactivated FBS and 6 µg/ml of polybrene for 48 h.

### 2.5 Flow cytometry analysis of cell apoptosis

The MC3T3‐E1 cells apoptosis were examined at 24 h after treatment. In accordance with the Annexin V‐PE/7‐AAD Apoptosis Detection Kit (Vazyme # A213‐01, China), 2 × 10^5^ cells were collected in each flow cytometry tube with 100 µl of PBS. Then, 5 µl of Annexin V binding buffer was added, followed by thorough mixing, and the tube was incubated in the dark at room temperature for 15 min. Next, 10 µl of 7‐AAD were added into the mix. The samples were analysed within 1 h using a FACScan flow cytometer (BD Biosciences). In this assay, cells in early apoptosis stain only with Annexin V via phosphatidylserine exposed in the membrane, cells in late apoptosis stain with both Annexin V and 7‐AAD and dead cells stain only with 7‐AAD.

### 2.6 Immunofluorescence analysis for autophagy

For immunofluorescence staining, MC3T3‐E1 cells were fixed with 10% formalin for 15 min, permeabilized with 0.25% Triton X‐100 (Beyotime #P0096, China) for 10 min in room temperature, then blocked in PBS with 5% bovine serum albumin for 30 min at room temperature. Immunostaining was performed using primary antibodies against LC3B (1:100 dilution, CST #3868, USA) and Beclin‐1 (1:100 dilution, Abcam ab210498, USA) and the secondary antibody against rabbit IgG (1:1000 dilution, CST #8889, USA). Nuclei were counterstained with 4’,6‐diamidino‐2‐phenylindole (DAPI, Solarbio #S2110, China). Images were captured using a fluorescence microscope (Leica TCS SPS, Germany) and were processed with Image J software (JAVA, USA).

### 2.7 Transmission Electron Microscopy (TEM)

After discarding the culture medium, MC3T3‐E1 cells were digested, sedimented by centrifugation and immediately mixed with 2.5% glutaraldehyde (Sigma Aldrich #G5882, USA) to crosslink the cellular components. After discarding the glutaraldehyde, the cells were centrifuged for 5 min at 250 g. Fresh glutaraldehyde was added, and the cells were then fixed in 2% OsO_4_ (Sigma Aldrich #20816‐20–1, USA). Samples were then dehydrated in a graded alcohol series, embedded in resin, cut into ultrathin sections and stained with uranyl acetate and citric acid. Autophagosomes were observed by transmission electron microscopy (JEOL JEM‐1400PLUS, Japan).

### 2.8 Western Blot (WB) analysis

Cells were lysed immediately in lysis buffer (62.5 mM Tris‐HCl, pH 6.8, 2% SDS, 10% glycerol, 50 mM dithiothreitol, 0.01% bromophenol blue, 1×protease inhibitor cocktail) for 10 min at 4°C. After centrifuge, the cell lysates were mixed with 4 × Laemmli Sample Buffer (Biorad #1610747, USA) for 5 min at 95°C. The mixed solution was analysed by SDS/PAGE and transferred electrophoretically to poly‐vinylidene fluoride (PVDF) membranes (Biorad #1620177, USA). The blot were blocked in 1% bovine serum albumins (Sigma Aldrich #A1933, USA) and then probed with antibodies against LC3B (1:1000 dilution, CST #3868, USA), Beclin‐1 (1:1000 dilution, Abcam #ab210498, USA), *p*‐AMPK (1:1000 dilution, CST #2535, USA), AMPK (1:1000 dilution, CST #5832, USA), cleaved caspase‐3 (1:1000 dilution, CST #9664, USA), caspase‐3 (1:1000 dilution, CST #9662, USA) or β‐actin (1:1000 dilution, CST #4970, USA) in 4°C overnight. After that the blot were probed with the secondary antibody against mouse (1:5000 dilution, Abcam ab6728, USA) or rabbit (1:5000 dilution, Abcam ab6721, USA) IgG and then processed using an enhanced chemiluminescence (ECL) kit (Santa Cruz, #SC‐2048, USA). The band intensity of each blot was quantified by Image J software (JAVA, USA) before normalizing to the corresponding loading controls (β‐actin), and the value was expressed as fold or percentage change from the loading control.

### 2.9 Statistical analysis

GraphPad Prism 7.0 software (San Diego, CA) was used for all statistical analyses. Data were presented as the mean ± standard error (SE). ANOVA was used for comparison among multiple groups, followed by pairwise comparisons using Tukey's post hoc test.

## 3 RESULTS

### 3.1 Apoptosis, autophagy and *p*‐AMPK levels are elevated in human SONFH specimens

**FIGURE 1:**
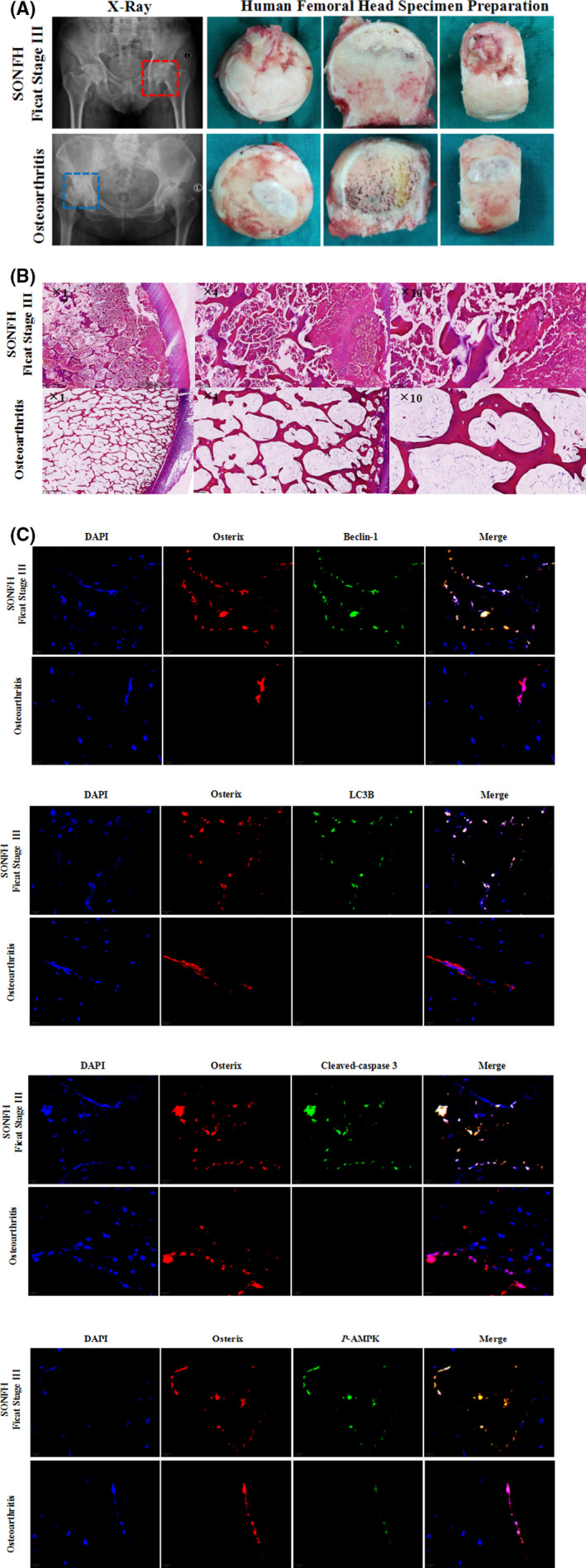
Apoptosis, autophagy and p‐AMPK levels are elevated in human SONFH specimens. (A) X‐ray and specimens of representative patients with Ficat stage III SONFH or Kellgren‐Lawrence stage IV hip osteoarthritis. (B) Representative H&E staining of SONFH (*n* = 5) and hip osteoarthritis (*n* = 5) specimens, showing necrotic bone and normal bone. (C) Representative image of Beclin‐1, LC3B, cleaved caspase‐3 and *p*‐AMPK expression in osteoblasts (Osterix) of SONFH (*n* = 5) and hip osteoarthritis (*n* = 5) specimens, based on immunofluorescence staining

Previous studies have shown that dexamethasone can increase apoptosis of osteoblasts and osteocyte in vivo or vitro.^5^, ^6^ Simultaneously, the intracellular level of autophagy increases, resisting apoptosis to a certain extent. To test the levels of apoptosis, autophagy and *p*‐AMPK in the SONFH specimens, we collected human SONFH and osteoarthritis patients femoral head (The demographic information were shown in Table S1), and used H&E to confirm the necrotic area (Figure 1A,B). We then performed immunofluorescence staining and investigated that the levels of autophagy (Beclin‐1, LC3B), apoptosis (cleaved caspase‐3) and *p*‐AMPK in osteoblasts (osterix) were significantly higher in SONFH than in osteoarthritis patients (Figure 1C). These indicated that the AMPK signalling pathway participates in the process of autophagy in the SONFH.

### 3.2 Aucubin within a certain concentration range prevents dexamethasone‐induced apoptosis of osteoblasts in a dose‐dependent manner

**FIGURE 2:**
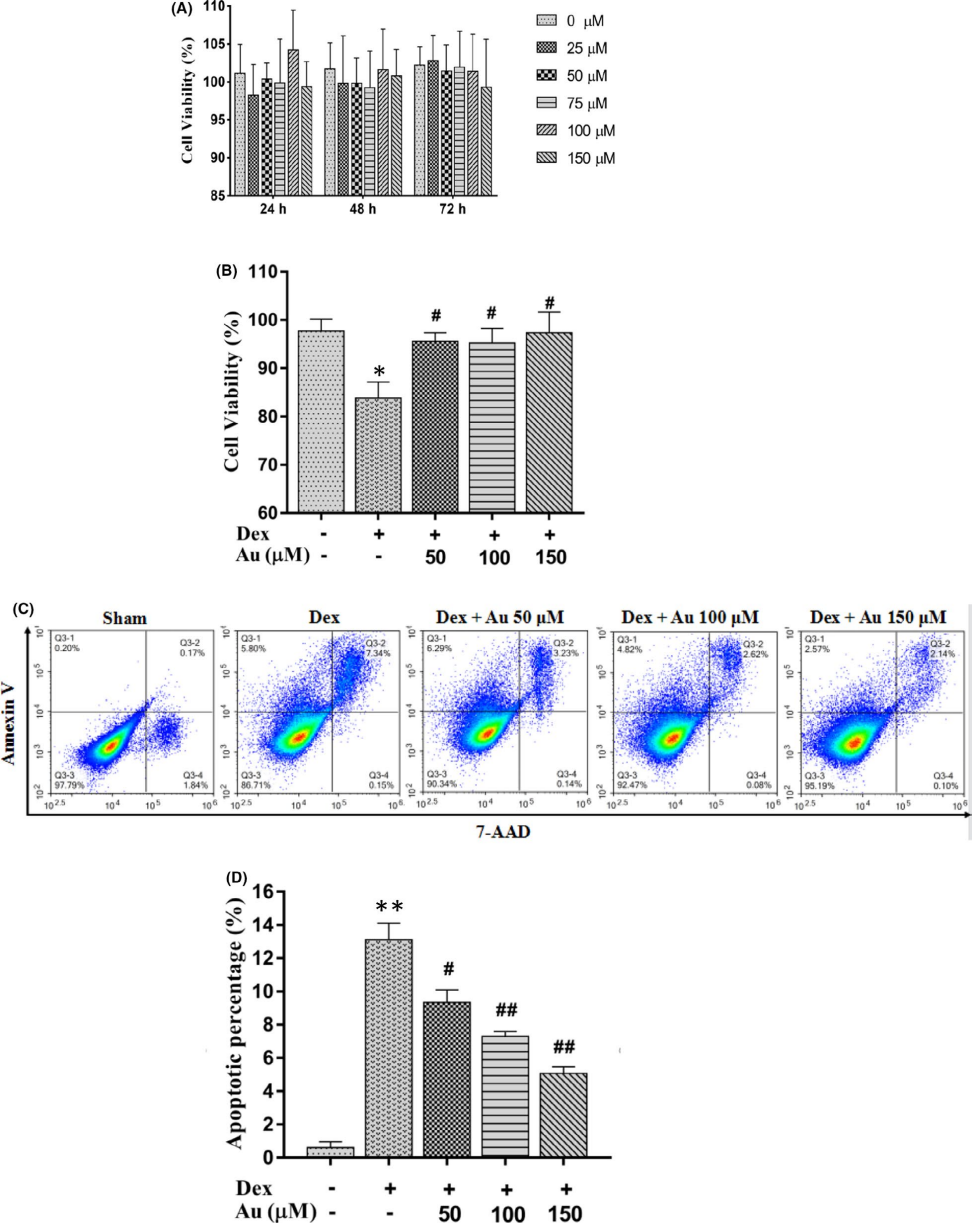
Aucubin within a certain concentration range prevents dexamethasone‐induced apoptosis of osteoblasts. (A) Quantification of MC3T3‐E1 cells viability under aucubin stimulation over time, based on the CCK‐8 test. (B) Quantification of MC3T3‐E1 cell viability in the presence of dexamethasone and various concentrations of aucubin, based on the CCK‐8 test. (C and D) Representative image and quantification of apoptosis in dexamethasone‐stimulated MC3T3‐E1 cells treated with different concentrations of aucubin, then analysed by flow cytometry. Dex indicates 1 µM dexamethasone; Au indicates aucubin. *, ** indicates *p* < 0.05, 0.01 when compared with sham group; #, ## indicates *p* < 0.05, 0.01 when compared with dexamethasone stimulation group

To explore the effect of aucubin on the dexamethasone‐induced apoptosis of osteoblasts, we first treated MC3T3‐E1 cells with a concentration gradient of aucubin. Concentrations from 25 μM to 150 μM did not show cytotoxicity (Figure 2A). Then, aucubin (50, 100 or 150 μM) and dexamethasone (1 μM) were co‐cultured with MC3T3‐E1 cells. After 48 h, the CCK‐8 assay and flow cytometry to measure cell viability found that aucubin effectively prevented dexamethasone‐induced apoptosis of MC3T3‐E1 cells in a dose‐dependent manner (Figure 2B–D). Those indicated that aucubin could prevent dexamethasone‐induced apoptosis of osteoblasts in a dose‐dependent manner.

### 3.3 Aucubin prevents dexamethasone‐induced apoptosis by enhancing autophagy in osteoblasts

**FIGURE 3:**
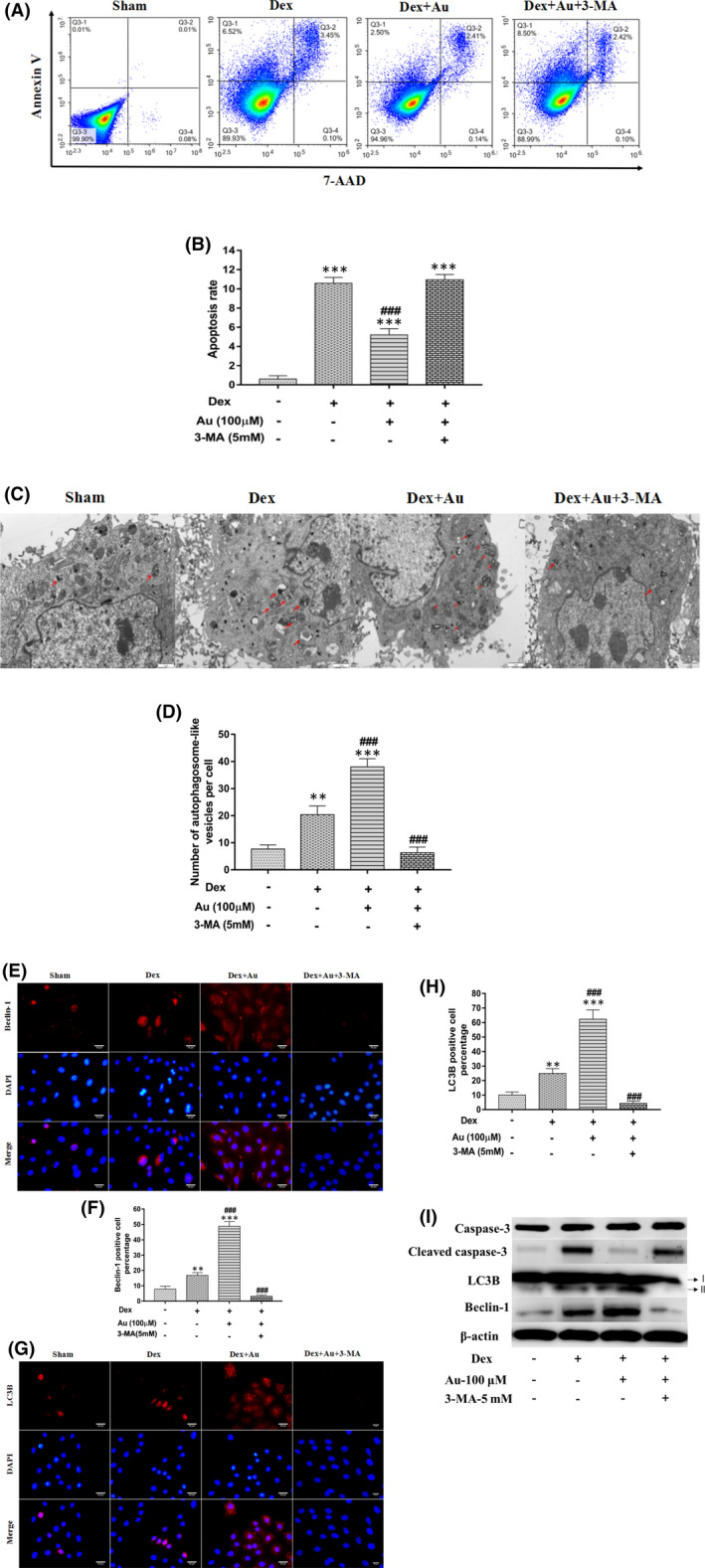
Aucubin prevents dexamethasone‐induced apoptosis by enhancing autophagy in osteoblasts. (A–B) Representative flow cytometry results and quantification of apoptosis in dexamethasone‐induced MC3T3‐E1 cells treated with aucubin, with or without 3‐MA treatment. (C–D) Representative images and quantification of autophagic vacuoles in dexamethasone‐induced MC3T3‐E1 cells treated with aucubin, with or without 3‐MA treatment, based on TEM (*n* = 5 in all groups). (E–H) Representative images and quantification of LC3B and Beclin‐1 levels in dexamethasone‐induced MC3T3‐E1 cells after treatment with aucubin, with or without 3‐MA treatment, based on immunofluorescence staining. (I) Representative images of LC3B, Beclin‐1 and cleaved caspase‐3 levels in dexamethasone‐induced MC3T3‐E1 cells after treatment with aucubin, with or without 3‐MA treatment, based on western blot. Dex indicates 1 µM dexamethasone; Au indicates aucubin. **, *** indicates *p* < 0.01, 0.001 when compared with Sham; ### indicates *p* < 0.001 when compared with dexamethasone stimulation

Aucubin has been shown to enhance autophagy and prevent apoptosis in several cell lines.^26^, ^27^ However, how aucubin prevents dexamethasone‐induced apoptosis in osteoblasts remains unclear. To assess its impact on autophagy, we treated dexamethasone‐stimulated MC3T3‐E1 cells with aucubin and found that aucubin prevented MC3T3‐E1 cells apoptosis, increased the number of autophagosomes and enhanced the expression of autophagy‐associated proteins LC3B and Beclin‐1 (Figure 3A–I, Figure S1). All these effects of aucubin were blocked by the autophagy inhibitor 3‐MA (Figure 3A–I, Figure S1). These data suggest that aucubin prevents dexamethasone‐induced osteoblast apoptosis by enhancing autophagy.

### 3.4 Aucubin enhances autophagy in osteoblasts by activating AMPK signalling

**FIGURE 4:**
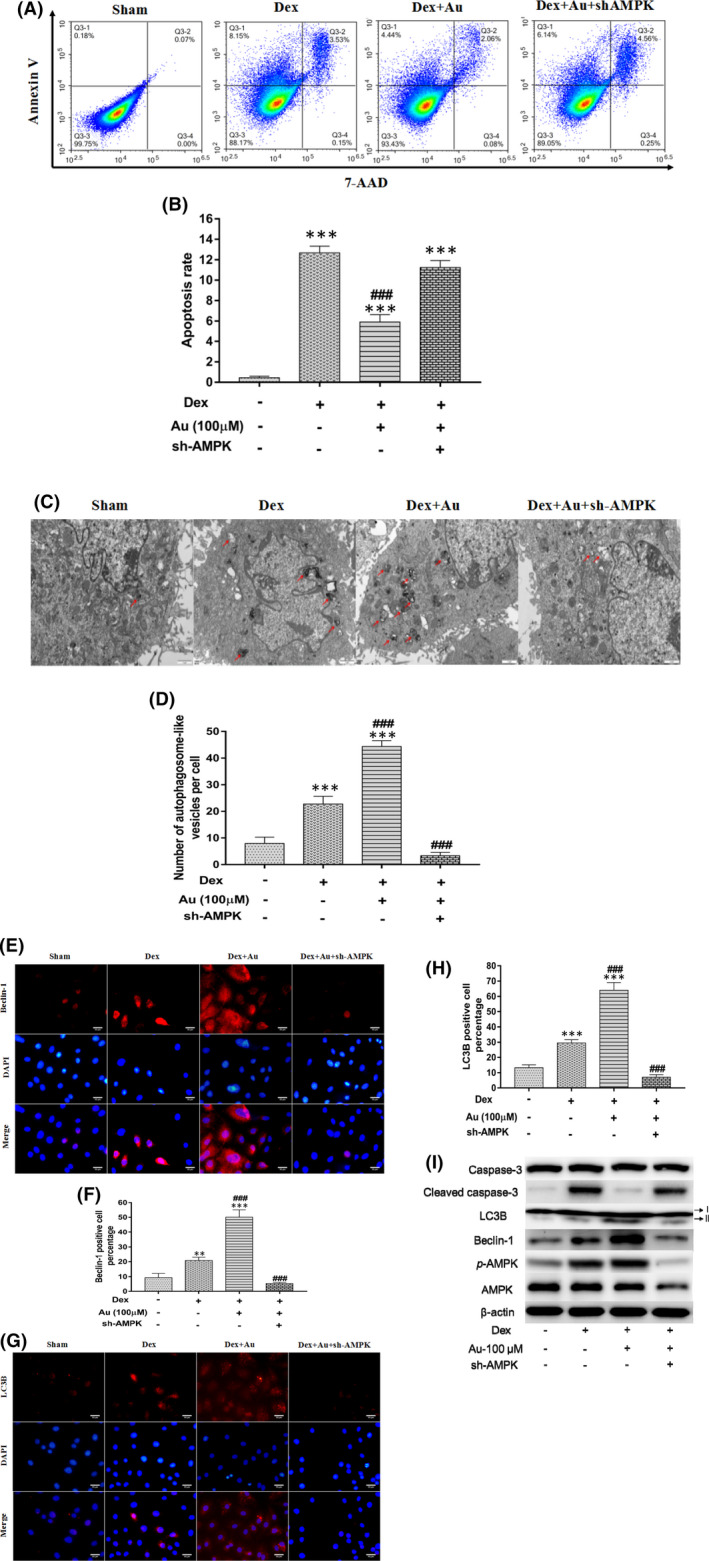
Aucubin enhances autophagy in osteoblasts by activating AMPK signalling. (A–B) Representative image and quantification of apoptosis via flow cytometry in dexamethasone‐induced MC3T3‐E1 cells treated with aucubin, with or without shAMPK (*n* = 5 in all groups). (C–D) Representative image and quantification of autophagic vacuoles in dexamethasone‐induced MC3T3‐E1 cells after aucubin, with or without shAMPK treatment, based on TEM (*n* = 5 in all groups). (E–H) Representative immunostaining micrographs and quantification of LC3B and beclin‐1 expression in dexamethasone‐induced MC3T3‐E1 cells after aucubin, with or without shAMPK treatment. (I) Representative Western blots image of *p*‐AMPK, AMPK, LC3B, Beclin‐1 and cleaved caspase‐3 in dexamethasone‐induced MC3T3‐E1 cells treated with aucubin, with or without shAMPK treatment. Dex indicates 1 µM dexamethasone; Au indicates aucubin. **, *** indicates *p* < 0.01, 0.001 when compared with sham; ### indicates *p* < 0.001 when compared with dexamethasone stimulation

Given that the AMPK signalling pathway can increase autophagy in cells and that *p*‐AMPK levels are increased in human SONFH specimens, we investigated whether aucubin upregulates AMPK signalling to enhance osteoblast autophagy under stimulation by dexamethasone. We knocked down AMPK in osteoblasts by infecting cells with lentivirus expressing shRNA (Figure S2), which partially reversed the ability of aucubin to decrease the levels of cleaved caspase‐3 and apoptosis rate (Figure 4A–B,I). The knockdown also led to disappearance of autophagic vacuoles and down‐regulated Beclin‐1, LC3B and *p*‐AMPK expression (Figure 4C–I, Figure S3). These results suggest that AMPK signalling helps mediate the ability of aucubin to increase autophagy and thus decrease dexamethasone‐mediated apoptosis in osteoblasts.

## 4 DISCUSSION

**FIGURE 5:**
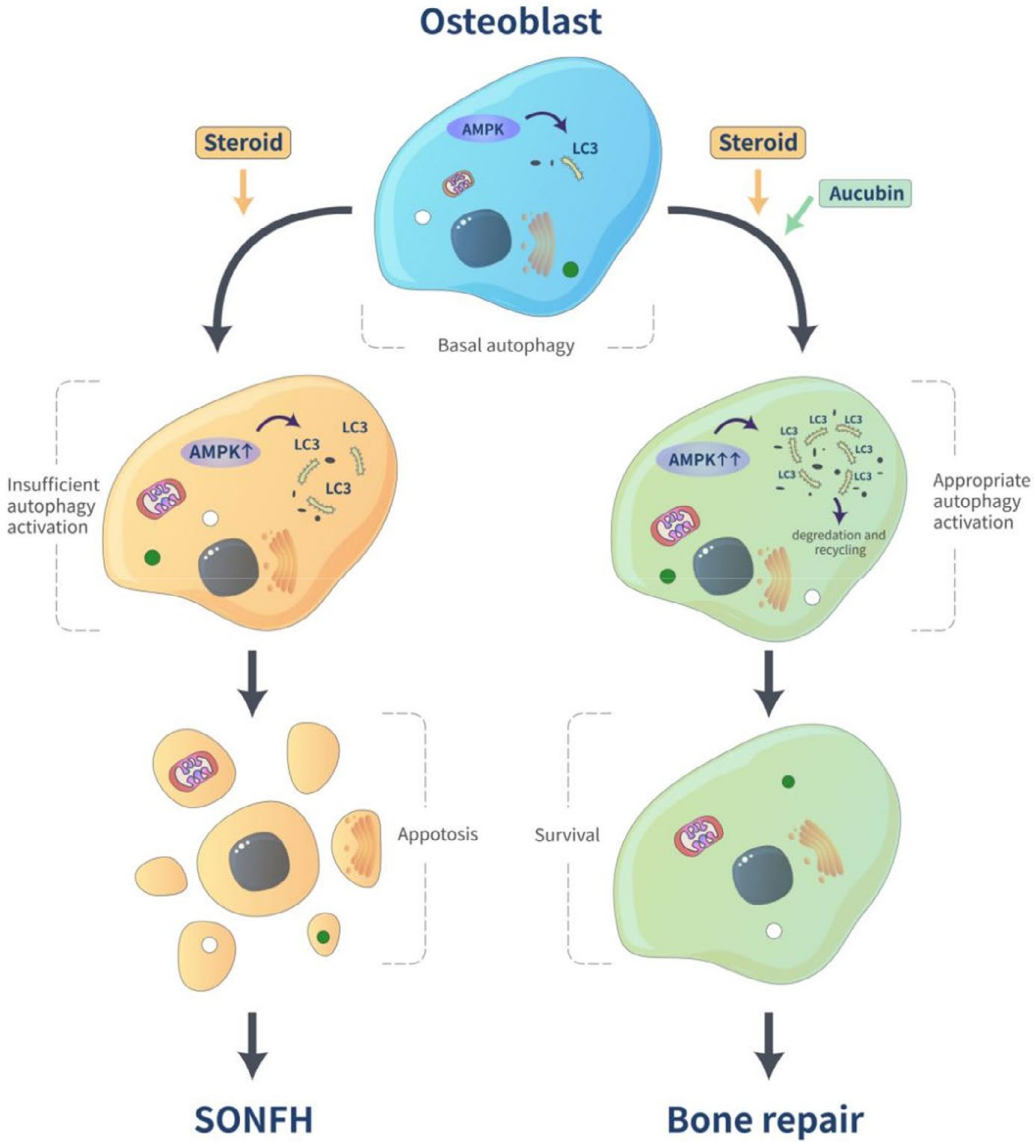
Schematic diagram showing the effect of aucubin on autophagy activation in dexamethasone‐induced osteoblasts: The autophagy of osteoblasts is maintained at basal levels under normal conditions and is increased when it faces excessive steroids to achieve self‐protection. However, insufficient autophagy activation fails to prevent osteoblasts apoptosis, eventually leading to osteonecrosis. Aucubin enhances the level of autophagy and effectively prevents steroid‐induced osteoblasts apoptosis by activating AMPK signalling

Aucubin can enhance autophagy to prevent cell apoptosis in several disease models.^26^, ^27^ Here, in this study, we were able to demonstrate that aucubin can counteract steroid‐induced osteoblast apoptosis by promoting AMPK‐dependent autophagy. The expression of *p*‐AMPK, LC3B and Beclin‐1 initially observed in human SONFH specimens suggested that AMPK signalling was involved in the regulation of autophagy in the pathogenesis of SONFH. Therefore, we hypothesized that AMPK signalling was integral to the mechanism of aucubin‐regulated autophagy. We designed an shRNA to knock‐down AMPK expression in osteoblasts and revealed that aucubin does indeed enhance osteoblast autophagy levels by activating AMPK signalling, which then prevents the apoptosis cascade normally triggered by dexamethasone stimulation. These findings provide evidence of a novel mechanism and a potential therapeutic strategy for SONFH (Figure 5).

Autophagy is an evolutionarily ancient mechanism. It is the major intracellular degradation system by which cytoplasmic materials are delivered to and degraded in the lysosome.^28^, ^29^, ^30^ The function of autophagy is not the simple elimination of materials, but a dynamic recycling system that produces new building blocks and energy for cellular renovation and homeostasis.^29^ Dysregulation of autophagy has been associated with the occurrence and development of many diseases covering almost every organ system.^30^, ^31^, ^32^ The autophagic process is maintained at basal levels under normal conditions, but is increased when cells face harmful factors and environmental stress in order to achieve self‐protection.^29^, ^31^, ^32^ Recent studies have linked the relationship between osteonecrosis and dysregulation of autophagy.^6^, ^28^ Inhibition of autophagy aggravates the negative effects of steroids on osteoblast proliferation, whereas activation of autophagy alleviates apoptosis. This indicates that activating autophagy is a cyto‐protective mechanism used by osteoblasts and bone marrow‐derived stem cells to oppose the cytotoxicity induced by excess steroid.^7^, ^32^ This literature and our present findings support the idea that the activation of autophagy is a novel and important strategy for the treatment of SONFH.

Many signalling molecules are involved in autophagy regulation, such as AMPK, mTOR, p53, p62 and the Bcl‐2 protein family.^29^, ^30^, ^31^, ^32^, ^33^, ^34^ AMPK is a highly conserved serine/threonine‐protein kinase, which acts as an intracellular energy sensor to activate catabolism and inactivate anabolism when the cellular AMP/ATP ratio increases.^15^, ^16^ In eukaryotes, AMPK has a critical role in regulating growth and metabolism, and has recently been linked to autophagy.^15^, ^16^, ^17^, ^18^, ^19^ AMPK may promote autophagy by directly phosphorylating autophagy‐related proteins in the mTORC1, ULK1 and PIK3C3/VPS34 complexes, or by indirectly regulating the expression of autophagy‐related genes downstream of transcription factors such as FOXO3, TFEB and BRD4.^17^ Previous studies have shown that activation of AMPK can promote autophagy by ameliorating osteoblast apoptosis and dysfunction induced by steroid or hydrogen dioxide.^33^, ^35^, ^36^, ^37^ Similarly, our present analysis of human SONFH specimens suggests that AMPK signalling participates in the pathology of SONFH. Therefore, targeted‐activation of AMPK to promote autophagy is a logical strategy to protect osteoblasts from steroid injury and to treat SONFH.

Natural herbal medicines are prominent in research and development of new drugs because of their typically minor side‐effects and low costs.^26^ *Eucommia ulmoides* Oliv. is the sole species of the genus *Eucommia*, and its leaf, stem, bark and staminate flower have been a source of traditional medicine in many Asian countries.^26^ In fact, *Eucommia ulmoides* Oliv. has been used in orthopaedics and traumatology for thousands of years as a bone‐strengthening herb. A total of 124 compounds are contained in *Eucommia ulmoides* Oliv., including the main active constituents of lignans and iridoids.^24^, ^38^, ^39^ Aucubin is one of the most important bioactive components and the most prevalent iridoid in *Eucommia ulmoides* Oliv.,^26^, ^38^ and it has been shown to reduce bone loss and apoptosis.^25^, ^39^ In this study, we observed that aucubin increased the level of autophagy and reduced the rate of apoptosis in steroid‐treated osteoblasts. These effects were partially reversed when AMPK was knocked down. Future work could investigate whether AMPK directly or indirectly regulates autophagy, such as by phosphorylating autophagy‐related proteins or regulating the expression of autophagy‐related genes downstream of transcription factors. A logical future direction of this work would include animal experiments and clinical trials to evaluate the efficacy of aucubin in preventing or slowing the progression of SONFH. Recently, several studies revealed a positive regulatory role of aucubin on AMPK signalling and autophagy in the hippocampus and cardiac fibroblasts,^26^, ^27^ indicating potential translation of our work into necrotic diseases in other organ systems as well.

## 5 CONCLUSION

In summary, our findings reveal that AMPK signalling participates in the regulation of autophagy in the pathogenesis of SONFH and aucubin can increase AMPK activation to promote autophagy and prevent steroid‐induced osteoblast apoptosis, laying a foundation for the further studies on the potential therapeutic role of aucubin in SONFH.

## CONFLICT OF INTEREST

The authors have declared that no competing interest exists.

## AUTHOR CONTRIBUTIONS

**Chen Yue:** Investigation (lead); Writing‐original draft (lead); Writing‐review & editing (equal). **Hongting Jin:** Investigation (equal); Methodology (equal); Project administration (equal); Writing‐original draft (equal); Writing‐review & editing (equal). **Xue Zhang:** Funding acquisition (equal); Investigation (equal); Software (equal); Writing‐original draft (equal); Writing‐review & editing (equal). **Wuyin Li:** Project administration (equal); Supervision (equal); Writing‐review & editing (equal). **Deli Wang:** Methodology (equal); Resources (equal); Writing‐review & editing (equal). **Peijian Tong:** Conceptualization (equal); Data curation (equal); Supervision (equal); Writing‐review & editing (equal). **Youwen Liu:** Conceptualization (equal); Formal analysis (equal); Funding acquisition (equal); Resources (equal); Writing‐review & editing (equal). **Zhen Tan:** Conceptualization (equal); Data curation (equal); Funding acquisition (equal); Writing‐review & editing (equal).

## Supporting information

Fig S1‐S3

Table S1

## Data Availability

Data are available upon reasonable request from the corresponding authors.
