# Supplementary material for: Aucubin prevents steroid‐induced osteoblast apoptosis by enhancing autophagy via AMPK activation
Source: J Cell Mol Med. 2021 Oct 6;25(21):10175–84. doi: 10.1111/jcmm.16954 (PMC8572759; doi:10.1111/jcmm.16954)
Supplement: Supplementary file 1 — Fig S1‐S3 [file JCMM-25-10175-s001.docx]

**Supplement Figure**


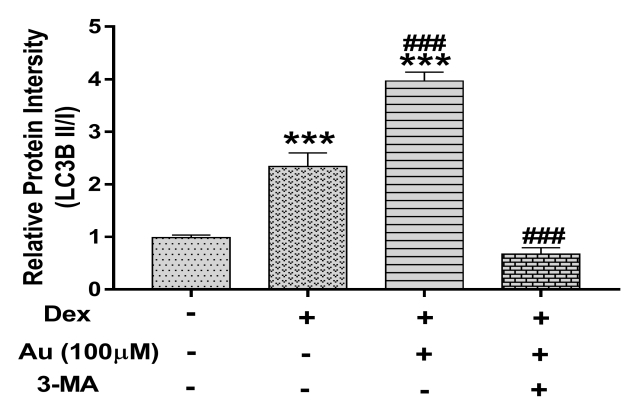

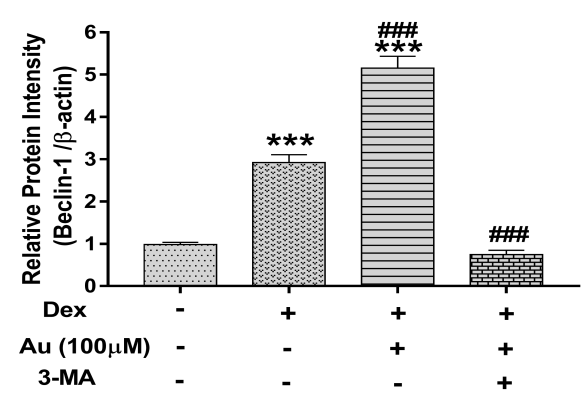

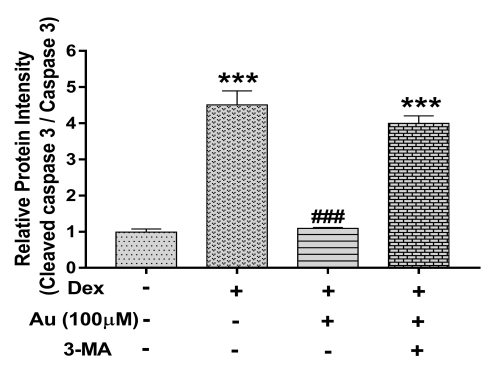


**Figure 1.** Quantification of the ratio LC3B II/I, Beclin-1 and cleaved caspase 3/ caspase 3 level in dexamethasone-induced MC3T3-E1 cells after treatment with aucubin, with or without 3-MA treatment, based on western blot. *** indicates p < 0.001 when compared with sham; ### indicates p < 0.001 when compared with dexamethasone stimulation.


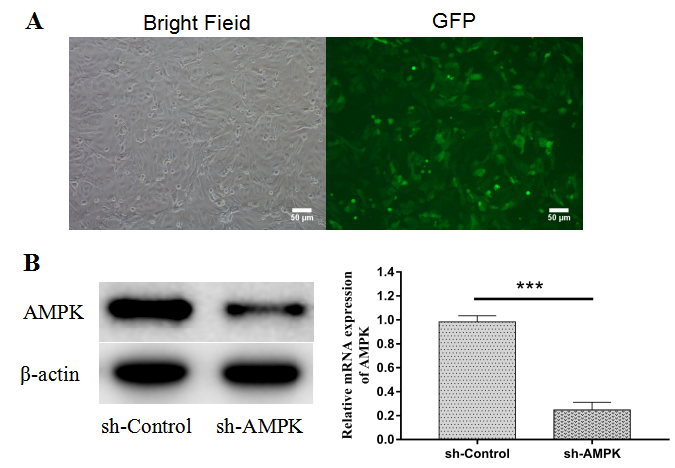


**Figure 2.** AMPK Knock-down lentiviral transduction. A: Representative images of MC3T3-E1 cells transfected with GFP-labeled sh-AMPK plasmid; B. Representative images of AMPK level in MC3T3-E1 cells based on Western Blot detection. *** indicates p < 0.01, 0.001 when compared with sham.


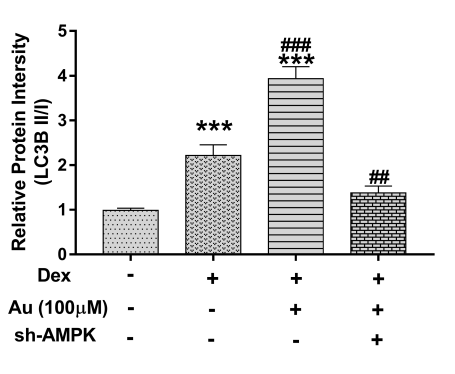

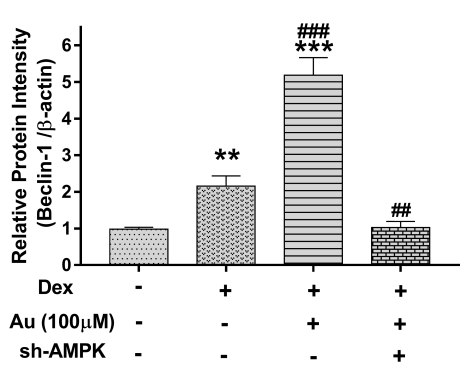

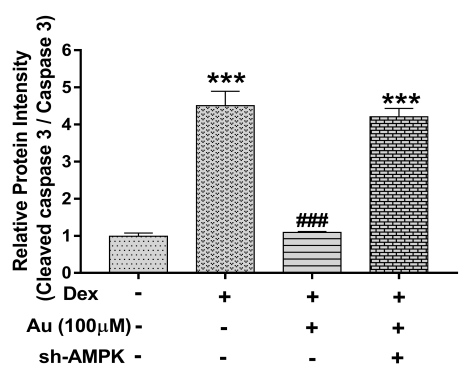

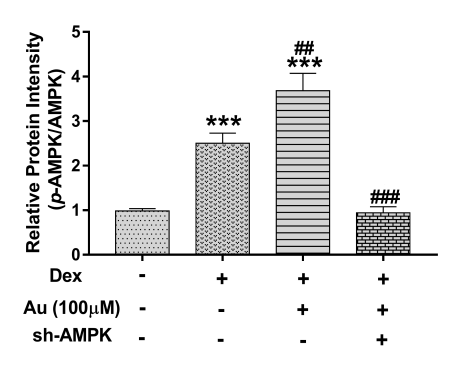


**Figure 3.** Quantification of the ratio LC3B II/I, Beclin-1, cleaved caspase 3/ caspase 3, and *p*-AMPK/AMPK level in dexamethasone-induced MC3T3-E1 cells treated with aucubin, with or without sh-AMPK treatment. **, *** indicates p < 0.01, 0.001 when compared with sham; ##, ### indicates p < 0.01, 0.001 when compared with dexamethasone stimulation.
