# Supplementary material for: Aucubin prevents steroid‐induced osteoblast apoptosis by enhancing autophagy via AMPK activation
Source: J Cell Mol Med. 2021 Oct 6;25(21):10175–84. doi: 10.1111/jcmm.16954 (PMC8572759; doi:10.1111/jcmm.16954)
Supplement: Supplementary file 2 — Table S1 [file JCMM-25-10175-s002.doc]

Supplementary Table 1: Basic information of patients

| Patient Number | Diagnosis | Age | Gender | Disease duration of HOA/SONFH | History of steroids application |
| --- | --- | --- | --- | --- | --- |
| 1 | HOA  (K-L III) | 65 | Female | 10 years | - |
| 2 | HOA  (K-L IV) | 48 | Female | 12 years | - |
| 3 | HOA  (K-L III) | 70 | Female | 25 years | - |
| 4 | HOA  (K-L IV) | 66 | Female | 30 years | - |
| 5 | HOA  (K-L III) | 54 | Male | 8 years | - |
| 6 | SONFH  (Ficat IV) | 42 | Male | 3.5 years | Oral prednison: 10mg qd for 6 months, 5mg qd for 1.5 years |
| 7 | SONFH  (Ficat III) | 48 | Female | 2 years | Oral prednison: 20mg qd for 3 months, 10mg qd for 6 months, 5mg qd for 6 months |
| 8 | SONFH  (Ficat III) | 53 | Male | 2 years | Methylprednisolone pulse therapy: 1g for 3 days; Oral methylprednisolone: 60mg qd for 1 months, and gradually reduce to 5mg qd |
| 9 | SONFH  (Ficat III) | 44 | Female | 3 years | Oral prednison: 10mg qd for 6 months, 5mg qd for 1 year |
| 10 | SONFH  (Ficat III) | 62 | Female | 1 year | Methylprednisolone pulse therapy: 500 mg for 7 days |

HOA: Hip osteoarthritis; K-L: Kellgren-Lawrence classification of osteoarthritis;

SONFH: Steroid-induced necrosis of femoral head; Ficat: Ficat classification of necrosis of femoral head
